# Metabolomic Profiling Reveals Potential Biomarkers and Prominent Features in HIV/AIDS Patients Co-Infected with SARS-CoV-2

**DOI:** 10.3390/microorganisms13010144

**Published:** 2025-01-13

**Authors:** Xuan Yan, Xinyu Zhang, Wei Song, Tangkai Qi, Zhenyan Wang, Yang Tang, Jianjun Sun, Shuibao Xu, Junyang Yang, Jiangrong Wang, Jun Chen, Renfang Zhang, Li Liu, Yinzhong Shen

**Affiliations:** Department of Infection and Immunity, Shanghai Public Health Clinical Center, Fudan University, Shanghai 201508, China; yanxuandr@163.com (X.Y.); zhangxinyu@shphc.org.cn (X.Z.); songwei@shphc.org.cn (W.S.); qitangkai@shphc.org.cn (T.Q.); wangzhenyan@shphc.org.cn (Z.W.); tangyang@shphc.org.cn (Y.T.); sunjianjun@shphc.org.cn (J.S.); xushuibao@shphc.org.cn (S.X.); yangjunyang@shphc.org.cn (J.Y.); wangjiangrong@shphc.org.cn (J.W.); chenjun@shphc.org.cn (J.C.); zhangrenfang@shphc.org.cn (R.Z.)

**Keywords:** HIV, SARS-CoV-2, untargeted metabolomics, lipid metabolism, metabolic biomarkers

## Abstract

The underlying mechanisms and diagnostic biomarkers for the progress of COVID-19 in HIV patients have not been fully elucidated. In this study, the aim is to analyze the metabolomic profiles of HIV/AIDS patients co-infected with SARS-CoV-2 and to identify biomarkers indicative of co-infection. In this study, we conducted a retrospective cohort analysis of peripheral blood samples collected from 30 HIV/AIDS patients co-infected with SARS-CoV-2 (pc group) and 30 patients without SARS-CoV-2 (nc group). In this study, through non-targeted metabolomics and lipidomics analysis, 77 differential metabolites were identified in the plasma of patients co-infected with HIV and SARS-CoV-2 compared to the nc group, with vitamin K1 emerging as a significant feature. Moreover, the plasma of the pc group showed disturbances in lipid metabolism, with elevated triglycerides (TG) and phosphatidylcholine (PC) and decreased phosphatidylglycerol (PG) compared to the control group. Vitamin K1 may be a biomarker for SARS-CoV-2 in HIV/AIDS patients, and changes in the levels of TG, PC, and PG molecules appear to be the main features following HIV co-infection with COVID-19. The emphasis in our study is on the power of using comprehensive metabolomics (lipidomics) approaches to identify metabolic biomarkers and potential mechanisms of COVID-19 in HIV/AIDS patients.

## 1. Introduction

The COVID-19 pandemic, caused by the novel coronavirus (SARS-CoV-2), led to millions of confirmed cases and deaths worldwide [1]. The Joint United Nations Programme on HIV/AIDS (UNAIDS) identified people living with HIV (PLWH)/AIDS as a high-risk group for COVID-19 [2]. HIV infection is a significant independent risk factor for severe cases and in-hospital deaths at the time of COVID-19 admission [3]. The rates of hospitalization, severe illness, and mortality due to COVID-19 are higher in patients with HIV/AIDS compared to those without [4,5]. However, when HIV/AIDS patients receive regular antiretroviral therapy (ART) and have good cellular immune status, their disease severity is not worse, and ART can reduce the incidence of adverse outcomes [6,7,8]. Studies have found that high HIV viremia and low CD4+ T cell counts are associated with poor prognosis in COVID-19, with a higher risk of adverse outcomes in HIV/AIDS patients with a CD4+ T cell count below 200 cells/μL and those who have not received ART [9,10,11].

HIV and SARS-CoV-2 originate from different viral families and exhibit significant differences in infection patterns, genomic content, and pathogenic mechanisms. However, both viruses promote the production of cytokines, which are associated with secondary complications in infected individuals [12,13]. Chronic cytokine release in HIV leads to ongoing inflammation, increased intestinal permeability, and bacterial translocation, which ART cannot reverse. Residual viral replication and other co-infections also prolong the inflammatory state [14]. COVID-19 patients may experience an acute cytokine response, including cytokine storms related to severe clinical symptoms and cardiac issues, affecting intestinal permeability and inflammation levels. An increase in systemic inflammation markers is a prognostic variable for COVID-19 [15]. Studies have also suggested that both viruses involve the formation of neutrophil extracellular traps (NETs), a mechanism of neutrophil death that can trigger cytokine storms and thrombotic complications in individuals infected with HIV or SARS-CoV-2 [16,17]. Substantial evidence indicates that both infections can cause significant changes in inflammation, gut microbiota, and coagulation.

Metabolomics and lipidomics have identified numerous circulating lipids directly associated with disease severity, making lipid metabolism a potential therapeutic target. These circulating lipids play a crucial role in the life cycle and pathogenesis of viruses, which can exploit lipid signaling and synthesis to influence the lipid metabolism of host cells. Therefore, understanding this interaction could reveal new ways of fighting viral diseases and developing treatments [18]. Individuals infected with HIV are prone to significant disruptions in blood lipids and lipoproteins, which are the result of both the infection itself, and the direct impact of drugs used to treat HIV on lipid metabolism [19]. Metabolomics has been used to study various aspects of HIV and SARS-CoV-2 infections, gaining a deeper understanding of the metabolic adaptations caused by infection and treatment. The mechanisms underlying autophagy in SARS-CoV-2-infected cells and the exosome-mediated cellular invasion by the virus have been elucidated through metabolomic analysis, shedding light on the metabolic dysregulation experienced by the organism following SARS-CoV-2 infection [18]. Lipidomics has been widely used to study the relative tendency of different antiretroviral drugs to alter lipid levels in HIV/AIDS patients [19]. Although metabolomics has contributed to these fields, it has not yet been systematically applied to describe the state of co-infection with HIV and SARS-CoV-2. Investigating the metabolic changes in COVID-19 in HIV patients, which are different from those in HIV patients who are negative for the novel coronavirus, and whether changes in metabolic complications will affect the progression and outcome of the disease through exacerbating disease progression and systemic inflammation, are very important for exploring the mechanisms of lipid metabolism and drug development [18].

In our study, we aimed to investigate the characteristic metabolic changes associated with COVID-19 in HIV patients. We examined the pathological upregulation or downregulation of metabolic substances, as well as the disrupted metabolic pathways, occurring in HIV patients who also contracted SARS-CoV-2 in comparison to those with HIV alone. This research enhances our understanding of the pathogenesis and progression of such co-infections, and it aids in the exploration of new diagnostic and therapeutic approaches, offering further insights.

## 2. Materials and Methods

### 2.1. Study Design

This investigation encompassed 30 cases of HIV patients co-infected with COVID-19 admitted to the Department of Infection and Immunity at the Shanghai Public Health Clinical Center (SPHCC) from February 2023 to March 2024, as the experimental group (pc group). Another 30 HIV/AIDS patients without COVID-19 were collected during the same time period as the control group (nc group) (a sample size of 30 per group is a common practice in metabolomics studies, which takes into account factors such as data analysis needs, sample heterogeneity, study costs, and case resources to ensure the reliability and validity of the study results). The inclusion criteria for the experimental group were as follows: (1) aged over 18 years old, (2) diagnosed with HIV cases in accordance with the diagnostic principles of the Chinese AIDS Diagnosis and Treatment Guidelines, and (3) confirmed cases of COVID-19 based on the diagnostic criteria set forth in the Diagnosis and Treatment Plan for COVID-19 (Trial Version 10), which includes a positive nucleic acid test for SARS-CoV-2. The inclusion criteria for the control group were as follows: (1) aged over 18 years old, (2) diagnosed with HIV cases in accordance with the diagnostic principles of the Chinese AIDS Diagnosis and Treatment Guidelines, and (3) negative for SARS-CoV-2 nucleic acid test. The exclusion criteria for both the experimental and control groups included the following: (1) pregnant women, (2) individuals with significant deviations from standard dietary practices, such as those following vegetarian or low-carbohydrate diets, (3) those suffering from severe malnutrition, active infections, or substance addiction, (4) patients diagnosed with irritable bowel syndrome, inflammatory bowel disease, or other gastrointestinal disorders, and (5) individuals with comorbidities involving severe diseases of the heart, brain, liver, kidneys, hematopoietic system, or other significant primary illnesses.

All participants’ peripheral blood samples in the pc group were collected on the first day of admission with a confirmed diagnosis of coinfection of HIV and SARS-CoV-2, and all participants’ peripheral blood samples in the nc group were collected on the first day of admission with a confirmed diagnosis of HIV infection. The samples were centrifuged at 2000 rpm for 10 min at 4 °C, and the supernatant was frozen in a −80 °C refrigerator. These plasma samples were then analyzed for untargeted metabolomics and lipidomics studies. Meanwhile, we also performed blood tests such as immune function and HIV viral load on the first day of the participants’ admission and collected the clinical indicators.

The protocol for this study passed the Ethics Committee of SPHCC (Ethics Approval Number: 2023-S082-01), with informed consent being obtained from all participants before collecting their peripheral blood.

### 2.2. Statistical Analysis

Statistical analyses were performed using Stata 16.0 and GraphPad Prism 8.3.1 software. Enumeration data were expressed as mean ± standard deviation (x ± s), and qualitative data were expressed as rate. The Chi-squared (χ^2^) test and the t-test were employed to identify statistically significant differences. Following sum normalization, the processed metabolic data underwent multivariate analysis using the R package ropls 1.38.0, which included pareto-scaled principal component analysis (PCA) and orthogonal partial least-squares discriminant analysis (OPLS-DA). The model’s reliability was confirmed through 7-fold cross-validation and response permutation testing. The variable importance in the projection (VIP) score for each variable in the OPLS-DA model indicated its influence on classification. Independent sample comparisons were evaluated for significance using Student’s *t*-test. A VIP > 1 and a *p* value < 0.05 were the criteria for identifying metabolites that showed significant changes. Based on the results of relative or absolute quantification of metabolites, the fold change (FC) in expression levels of a particular metabolite between two groups was calculated. A correlation analysis using Pearson’s method was conducted to ascertain the relationship between two variables.

## 3. Results

### 3.1. Study Cohorts

In line with our predefined inclusion and exclusion criteria, the pc group initially screened 40 HIV/AIDS patients co-infected with SARS-CoV-2. However, 10 patients were subsequently excluded because of intestinal diseases and severe renal failure. Similarly, the nc group started with 58 HIV/AIDS patients, but 28 were excluded due to liver and renal injuries. Table 1 presents the brief clinical background of the subjects involved in this study. A total of six participants in the pc group and two in the nc group had undetectable HIV viral loads. In the group of HIV/AIDS patients co-infected with SARS-CoV-2, there were nine cases of mild COVID-19, thirteen cases of moderate COVID-19, two cases of severe COVID-19, and six cases of critical COVID-19. All participants were discharged from the hospital after a period of treatment when their symptoms had significantly subsided. We attempted to match the main risk factors associated with HIV co-infection with SARS-CoV-2, including age, HIV RNA, CD4+ T cell count, lymphocyte count, and the status of ART. Following the application of χ^2^ tests and *t*-tests, these factors were found to be statistically insignificant between the HIV/AIDS patients co-infected with SARS-CoV-2 and those without SARS-CoV-2 infection (*p* > 0.05).

### 3.2. Metabolic Characteristics of Plasma Samples from Two Groups

**Figure 1:**
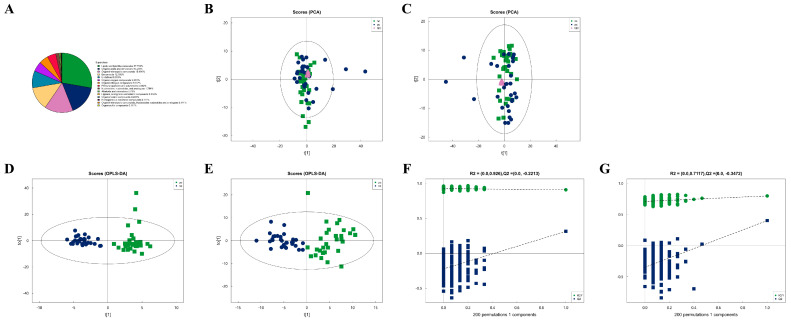
Metabolic characteristics of plasma samples from two groups. (**A**) Percentage of identified metabolites in each chemical classification. Metabolites with no chemical classification attribution were defined as undefined. (**B**) The PCA model performed on the entire sample set including pc, nc, and QC samples of positive ion mode, t[1] represents principal component 1 and t[2] represents principal component 2, each dot represents the plasma metabolomic profile of a single sample. (**C**) The PCA model performed on the entire sample set including pc, nc, and QC samples of negative ion mode. (**D**) OPLS-DA score plot of pc and nc of positive ion mode, t[1] represents principal component 1 and to[1] represents principal component 2, the ellipse represents the 95% confidence interval. The dots of the same color represent each biological duplication within a group, and the distribution status of the dots reflects the degree of variation between and within groups. (**E**) OPLS-DA score plot of pc and nc of negative ion mode. (**F**) OPLS-DA permutation test of positive ion mode. The horizontal coordinate represents the permutation retention, that is, the proportion in the same order as the original model Y variable, and the vertical coordinate represents the values of R^2^ and Q^2^. The two dashed lines represent the regression lines of R^2^ and Q^2^. R^2^ and Q^2^ in the upper right corner indicate that the permutation retention is equal to 1. (**G**) OPLS-DA permutation test of negative ion mode.

After combining both positive and negative ion modes, a total of 897 metabolites were identified, with 519 metabolites detected in the positive ion mode (Pos) and 378 in the negative ion mode (Neg). As shown in Figure 1A, all identified metabolites were categorized and counted according to their chemical taxonomy, revealing that lipids and lipid-like molecules constituted the largest proportion. PCA was performed to survey the metabolomics dataset and to discern the characteristics of the two groups. As depicted in Figure 1B,C, the quality control (QC) samples, which were a mixture of equal parts from each group and are shown as purple dots, clustered in the center, indicated good instrumental repeatability and stability throughout this study. An OPLS-DA model was utilized to further investigate metabolic changes and differential metabolites. In Figure 1D,E, the nc and pc groups are well separated, with high intra-group cohesion and distinct inter-group separation, demonstrating the reliability of the model and significant differences between the groups. Following a 7-fold cross-validation, the parameters for explained variation (R^2^), an indicator of model robustness, and cross-validated predictive ability (Q^2^) were determined to be as follows: in positive ion mode, a cumulative R^2^ of 0.91 and a Q^2^ of 0.316; in negative ion mode, a cumulative R^2^ of 0.8 and a Q^2^ of 0.404. To prevent overfitting in the supervised model during the modeling process, the reliability of the model was assessed using a permutation test. As shown in Figure 1F,G, as the degree of permutation retention decreased, both the R^2^ and Q^2^ values for the random models gradually declined, indicating that the original model did not exhibit overfitting and that the model’s stability was good.

### 3.3. Difference Analysis of Metabolites Between Two Groups and Hierarchical Clustering Analysis (HCA) of Differential Metabolites

**Figure 2:**
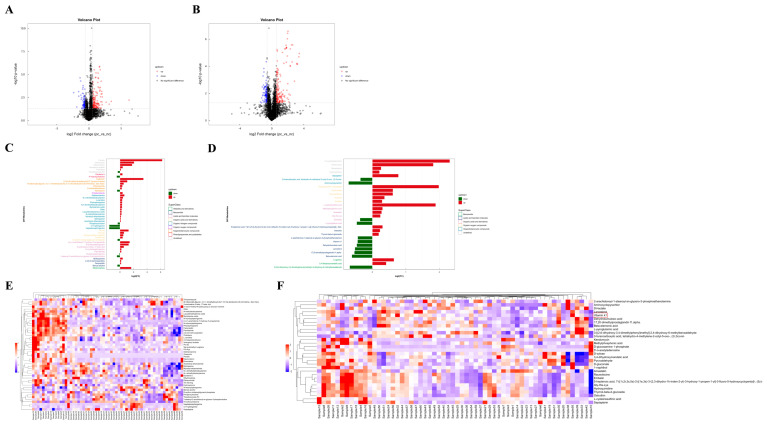
Difference analysis of metabolites between two groups and hierarchical clustering analysis (HCA) of differential metabolites. (**A**) Volcano plot visualization of positive ion mode. (**B**) Volcano map visualization of negative ion mode. (**C**) Analysis of different fold of significantly changed metabolites of positive ion mode. (**D**) Analysis of different fold of significantly changed metabolites of negative ion mode. (**E**) Heatmap visualization of hierarchical clustering of significantly changed metabolites of positive ion mode. Samples 1 to 30 are in the pc group, samples 31 to 60 are in the nc group. (**F**) Heatmap visualization of hierarchical clustering of significantly changed metabolites of negative ion mode. Samples 1 to 30 are in the pc group, samples 31 to 60 are in the nc group. The red box shows differential metabolite of concern.

Based on univariate analysis, a differential analysis was conducted on all metabolites detected under positive and negative ion modes, including those not identified. Using the nc group as the control, metabolites with an FC > 1.5 or <0.67 and a *p* value < 0.05 were selected. These metabolites were visually presented using volcano plots, as shown in Figure 2A,B. The metabolite features with OPLS-DA VIP values > 1.0 and *p* value < 0.05 were used as criteria for screening significantly differential metabolites, which were considered potential differential metabolites. As summarized in Table 2 and Table 3, there were 47 significantly differential metabolites of positive ion mode and 30 of negative ion mode. Interestingly, lipids constitute the largest proportion of these differential metabolites. The changes in the fold differences of the identified differential metabolites were intuitively displayed using bar charts, as shown in Figure 2C,D.

To more comprehensively and intuitively display the relationships between samples and the differences in expression patterns of metabolites across various samples, the expression levels of all samples and differential metabolites were normalized by subtracting the mean value of their respective groups and then dividing by the square root of the group’s standard deviation. Following this normalization, a distance matrix was calculated, and HCA was performed. The significantly differential metabolites (VIP > 1, *p* value < 0.05) were subjected to hierarchical clustering analysis, with the results shown in Figure 2E,F. Metabolites that cluster together exhibit similar expression patterns, suggesting they may have similar functions or be involved in the same metabolic processes or cellular pathways.

### 3.4. Vitamin K1 May Be a Potential Metabolic Biomarker for COVID-19 in HIV Patients

**Figure 3:**
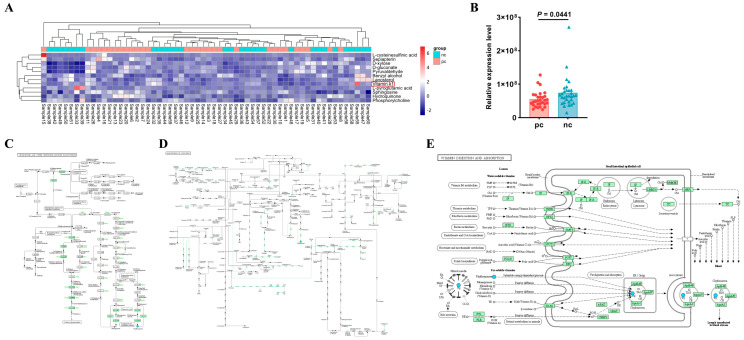
Vitamin K1 may be a potential metabolic biomarker for the development of HIV Co-infected with SARS-CoV-2. (**A**) Heatmap visualization of hierarchical clustering of significantly changed metabolites in the KEGG pathway. Samples 1 to 30 are in the pc group, samples 31 to 60 are in the nc group. The red box shows differential metabolite of concern. (**B**) Differences in relative expression level of vitamin K1 in KEGG pathway between pc and nc groups. The KEGG pathway mapper based on differential metabolic pathways involved in vitamin K1 between the nc group and the pc group includes (**C**) ubiquinone and other terpenoid-quinone biosynthesis, (**D**) biosynthesis of cofactors, and (**E**) vitamin digestion and absorption. In the pathway diagrams, metabolites are represented by circles. Upregulated metabolites (FC > 1.00, *p* value < 0.05) are indicated by red background circles, while downregulated metabolites (FC < 1.00, *p* value < 0.05) are indicated by blue background circles.

After merging the differential metabolites identified in both positive and negative ion modes, we utilized the Kyoto Encyclopedia of Genes and Genomes (KEGG, http://www.kegg.jp/ (accessed on 29 April 2024)) database for pathway annotation and analysis. To facilitate the observation of the expression of each differential metabolite annotated in the KEGG metabolic pathways, we selected KEGG metabolic pathways with more than two differential metabolites and presented them in the form of a heatmap (Figure 3A). We found that in the metabolic process, the expression level of vitamin K1 in HIV/AIDS patients co-infected with SARS-CoV-2 was lower than that in HIV/AIDS patients without SARS-CoV-2 infection (Figure 3B), with a statistically significant difference (*p* value < 0.05). Using the KEGG pathway mapper function, we displayed the differential metabolic pathways involving vitamin K1, including the ubiquinone and other terpenoid–quinone biosynthesis (Figure 3C), the biosynthesis of cofactors (Figure 3D), and vitamin digestion and absorption (Figure 3E). Collectively, our data suggest that vitamin K1 plays a significant role in HIV/AIDS patients co-infected with SARS-CoV-2 and could potentially be used to differentiate between HIV/AIDS patients co-infected with SARS-CoV-2 and those without SARS-CoV-2 infection. The metabolic pathways involved may represent the mechanisms by which vitamin K1 influences the occurrence and development of HIV co-infection with COVID-19.

### 3.5. Lipid Metabolic Characteristics of Plasma Samples from Two Groups

**Figure 4:**
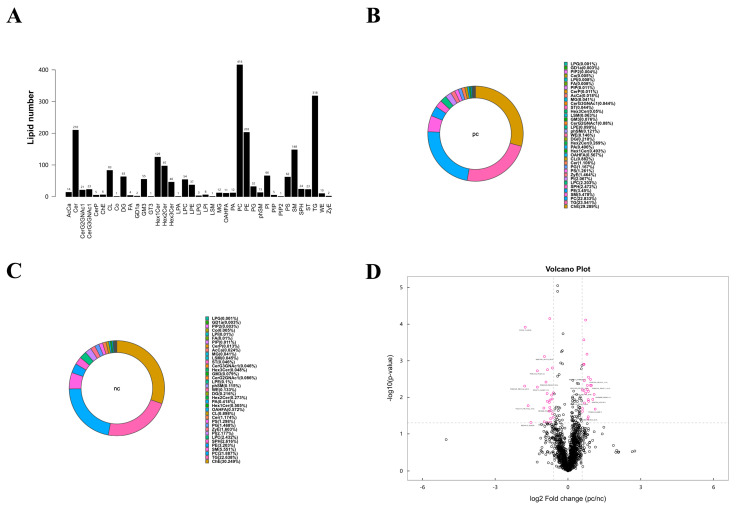
**Lipid metabolism characteristics of plasma samples from two groups.** (**A**) Statistical chart of lipid classes and the number of lipid species in the two groups. (**B**) Lipid class composition of the pc group. (**C**) Lipid class composition of the nc group. (**D**) Volcano plot visualization of significantly changed lipid species. Lipid molecules that meet FC > 1.5, *p* value < 0.05 or FC < 0.67, *p* value < 0.05 are indicated in rose red, while non-significantly different metabolites are shown in black. The TOP 10 with upregulated expression change (FC) and the TOP 10 with downregulated expression change (FC) were selected for labeling.

According to the results of the metabolomics analysis, we found that changes in lipid metabolism seem to play an important role in the development of HIV/AIDS patients co-infected with SARS-CoV-2. Therefore, we conducted an absolute quantitative lipidomics analysis with high-resolution on the plasma samples from the patients. The total content of lipid molecules in the same sample was obtained by summing up the content of all quantified lipid molecules. Our study identified a total of 39 lipid classes and 2216 lipid species of both positive and negative ion modes (Figure 4A). The lipid composition of the two groups differed, but both were predominantly composed of serum choline esterase (Figure 4B,C). Based on univariate analysis, we performed a differential analysis on all detected lipid molecules and presented the results in the form of a volcano plot. Lipid molecules with an FC > 1.5 or FC < 0.67 and *p* value < 0.05 were represented in different colors, and the top 10 upregulated and downregulated FCs in expression were selected and marked (Figure 4D). We used the OPLS-DA model to further investigate metabolic changes and differential metabolites, with the criteria of OPLS-DA VIP > 1 and *p* value < 0.05 to screen for differential lipid molecules that significantly contributed to the model interpretation (Table 4).

### 3.6. Targeted Metabolomic Analysis of Lipid Metabolic Pathways: Focus on TG, PG, and PC Classes

**Figure 5:**
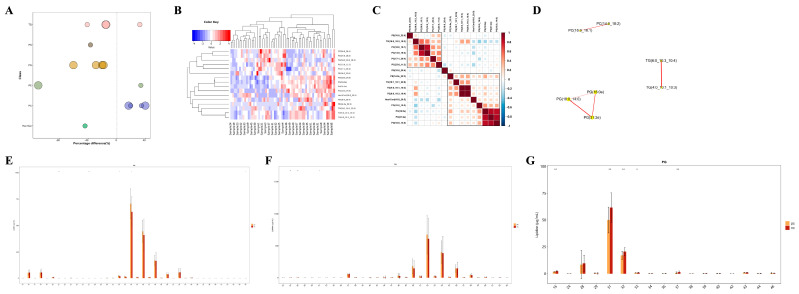
**Targeted metabolomic analysis of lipid metabolic pathways: focus on TG, PG and PC classes.** (**A**) Bubble map visualization of significantly different (VIP > 1, *p* value < 0.05) lipid species of different lipid classes. The bubbles in the plot represent significantly different lipid species. The vertical coordinates represent the lipid classes, which are distinguished by different colors. The bubble size represents the significance of the differences, with smaller bubbles representing significant differences (0.01 < *p* value < 0.05) and larger bubbles representing highly significant differences (*p* value < 0.01). (**B**) Cluster heatmap visualization of significantly different lipid species. Different color blocks represent the relative expression levels of lipid species, and lipid species with similar expression patterns are clustered in the same cluster on the left. (**C**) Heatmap visualization of correlation clustering. Red represents positive correlation, blue represents negative correlation, and the color depth is related to the absolute value of the correlation coefficient. The higher the degree of positive or negative correlation, the darker the color. (**D**) Network diagram of significantly different lipid species. The dots in the diagram represent significantly different lipid species, and the size of the dots correlates with the degree of connectivity; the larger the degree, the larger the dots. The color of the line represents the correlation, with red representing positive correlations and blue representing negative correlations. The thickness of the line represents the absolute value of the correlation coefficient, the thicker the line, the greater the correlation. (**E**–**G**) Distribution of carbon chain lengths of lipid species of PC, TG, PG. The horizontal coordinate shows the lipid species of different carbon chain lengths, and the vertical coordinate shows the content of lipid species. * *p* < 0.05, ** *p* < 0.01.

Unlike polar metabolites such as amino acids and nucleotides, the functional study of lipids is primarily conducted on the basis of classes, with different lipid classes having distinct biological functions [20]. Changes in the content of lipid classes can reflect changes in lipid function. Therefore, by comparing the expression changes in lipid classes in different samples, it is possible to screen for important lipid classes that may be involved in relevant biological processes. In this study, we visually presented the significantly differential lipid molecules (VIP > 1, *p* value < 0.05) using a bubble chart (Figure 5A), and found that the TG, PG, and PC classes contained extremely significantly differential lipid molecules (*p* value < 0.01). Moreover, HIV patients co-infected with SARS-CoV-2 had higher levels of TG and PC, and lower levels of PG. To assess the validity of the differential lipids, we performed hierarchical clustering on the samples using the expression levels of significantly differential lipids (VIP > 1, *p* value < 0.05) (Figure 5B). In general, when the candidate lipids selected are reasonable and accurate, samples from the same group should appear in the same cluster through clustering. Additionally, lipids within the same cluster have similar expression patterns, possibly indicating that they are involved in closely related metabolic steps. Subsequent correlation analysis of the significantly differential lipids revealed that there were significantly correlated lipid molecules within the TG, PG, and PC classes (Figure 5C,D), suggesting that they may be functionally related and involved in the same or closely related metabolic steps. Apart from the content of lipids, the chain length of lipids is also an important factor, as lipids have chain-specific physiological and pathological properties. In our study, it was found that PC lipid molecules with 32 or 34 carbon atoms, TG lipid molecules with 24 or 26 carbon atoms, and PG lipid molecules with 31 or 32 carbon atoms showed significant differences in content between the two groups (Figure 5E–G), indicating changes in the function and metabolism of lipids in HIV patients co-infected with COVID-19 from those without SARS-CoV-2 infection.

## 4. Discussion

In this study, after matching the main risk factors associated with HIV co-infection with SARS-CoV-2, we analyzed the plasma of PLWH to see how SARS-CoV-2 co-infection affects their metabolism. Subsequent analysis indicated that the plasma of the two groups was well distinguished, implying that the metabolic profile of HIV co-infection with SARS-CoV-2 underwent unique changes. A total of 77 metabolites were identified as differential metabolites to differentiate between HIV patients without SARS-CoV-2 co-infection and those co-infected with SARS-CoV-2. Analysis of these potentially altered metabolites and metabolic pathways revealed that vitamin K1 and its associated metabolic pathway disorders were among the most significant metabolic features. Non-targeted metabolomic analysis also found that lipid metabolites not only accounted for the largest proportion in both groups but also showed more significant changes in content among HIV/AIDS patients with SARS-CoV-2 co-infection. We further evaluated this using high-resolution non-targeted lipidomics absolute quantification analysis and found that TG, PG, and PC lipid classes were closely related to HIV co-infection with SARS-CoV-2. Several lipid molecules under these three classes showed extremely significant statistical differences between the two groups, such as TG (4:0_10:1_10:3) +Na, PG (31:2e) +NH_4_, PC (16:0_16:1) +HCOO, etc. Specific lipid species and those with varying carbon chain lengths showed notable differences, suggesting they could be biomarkers for COVID-19 in PLWH. Studying the metabolic pathways in which these differential metabolites participate and deducing the regulatory enzymes and genes involved can help reveal their mechanisms in life activities and complete research on regulatory pathways.

Retroviruses and coronaviruses have spread from animal hosts to humans, causing two ongoing pandemics and the death of tens of millions of people worldwide. The spread of both viruses in the population has been complicated by the emergence of drug-resistant mutants or other variants, which also have the potential to undermine the immunity gained from COVID-19 vaccination and previous infections [21]. During SARS-CoV-2 infection, there is a sharp decrease in the total number of CD4+ T cells, leading to immune dysfunction in the body [22]. Interestingly, both HIV-1 and SARS-CoV-2 infections lead to a decrease in CD4+ T cell counts, and the pro-inflammatory environment caused by HIV and SARS-CoV-2 is very similar [23]. Studies have shown that the mortality rate of COVID-19 in PLWH is 2.4 times higher than in the HIV-negative population [24]. Research has outlined the importance of biomolecular condensates (BMCs) in the replication cycles of HIV-1 and SARS-CoV-2 [21]. Studies have also shown that Neuropilin-1 (NRP-1), a type I transmembrane protein, serves as a receptor for the coronavirus infection of cells. The S1 protein of the novel coronavirus can interact with the host cell surface receptor NRP-1 to guide the virus into the cell, thereby promoting coronavirus infection. However, NRP-1, as a bone marrow cell-specific protein, is an inhibitor of HIV-1 infectivity and has anti-HIV activity [25]. These studies indicate that there are indeed potential biomarkers during co-infection with HIV and SARS-CoV-2. However, these targets have not yet fully elucidated the progression and mechanisms of HIV co-infection with SARS-CoV-2. Metabolomics has garnered increasing attention in virus research [26]. Lipidomics, a branch of metabolomics, is considered a powerful technique for systematically studying all lipids and lipid metabolism-related pathways. Our study reveals the potential and feasibility of using metabolomics to analyze diseases co-infected with HIV.

In our study, we found differences in the levels of vitamin K1 in the plasma of HIV patients who tested negative versus positive for SARS-CoV-2 nucleic acid. Studies have indicated that exogenous vitamin K1 can prevent and mitigate inflammation induced by lipoproteins both in vitro and in vivo. Moreover, vitamin K1 and its derivatives can prevent lipid peroxidation and exert anti-inflammatory effects [27,28]. Additionally, vitamin K is well-known for its role in promoting and dissolving blood clots, and a deficiency in vitamin K leading to impaired anticoagulant activity is one of the causes of microvascular thrombosis in COVID-19 [29]. Research has found low levels of vitamin K in comorbidities associated with poor prognosis in COVID-19 [9]. Lung inflammation-induced extrahepatic vitamin K deficiency can lead to the degradation of elastic fibers and accelerated thrombosis [29]. Persistent systemic immune activation, microvascular, and macrovascular diseases following HIV infection seem to lead to continuous abnormalities in coagulation function, which in turn appears to trigger an increased risk of complications, including but not limited to venous and arterial thrombotic diseases [30]. It is thus plausible to suggest that vitamin K1 could be a viable supplement in the treatment regimen for severe infectious diseases, given its potential to exert anti-inflammatory effects and protect against microvascular lesions. Based on our current research, it is not sufficient to clarify whether the lower expression of vitamin K1 in HIV patients who test positive for COVID-19 is due to the action of the novel coronavirus or is related to inflammation activation and immune abnormalities in HIV patients, and further in-depth research is needed. Subsequent research on vitamin K1 should aim to encompass larger study sample sizes, rigorously assess the impact of confounding variables, and construct prognostic models. These efforts will be instrumental in elucidating the diagnostic potential and clinical prognostic significance of vitamin K1 in the context of HIV and SARS-CoV-2 co-infection.

In addition, in our study, 16 lipid molecules were identified with significantly differential expressions, primarily from the TG, PC, and PG classes. Furthermore, through correlation analysis, we found that different differential lipid molecules within the same class are interrelated. Viruses exploit the host’s lipid metabolic mechanisms to achieve efficient replication [31]. Under different treatment conditions or biological processes, the lipid composition changes accordingly, and by analyzing the lipid composition, one can examine the main lipid composition and content distribution range of the samples as a whole. Different levels of lipid structure correspond to different levels of lipid function. Lipids often exert their functions in groups by class, so lipid function research usually focuses more on changes at the class level, while biomarker research typically concentrates on the expression levels of individual lipid species and their diagnostic capabilities [20]. HIV infection involves a series of important interactions with lipid components in the host membrane, which can regulate binding, fusion, internalization, and viral assembly. Existing data indicate that HIV actively alters the composition and content of lipids in the cell membrane to create an environment conducive to infection [32]. SARS-CoV-2 infects endothelial cells, causing endothelitis and damaging the endothelial layer. Endothelial cells have the capacity to metabolize several important lipids, including the biosynthesis of eicosanoids and platelet-activating factors, which are likely to be important physiological functions of endothelial cells. These potent lipids appear to play a role in maintaining vascular tone and mediating interactions between the endothelium and circulating inflammatory cells [33,34,35]. SARS-CoV-2 infection is linked to a pronounced pro-inflammatory cytokine profile, which likely plays a significant role in the liver abnormalities observed [36]. Studies on COVID-19 patients have found that they often have dyslipidemia, which is associated with an increased risk of severe outcomes. The downregulation of lipids appears to inhibit viral replication, suggesting their involvement in the viral replication and pathogenic processes, and highlighting their potential as targets for drug development [37,38]. We infer that on the basis of the HIV virus disrupting the lipids of host cells, SARS-CoV-2 has damaged the vascular endothelial cells and liver metabolism, thereby manifesting as abnormal lipid metabolism.

Triglycerides serve as a significant source of cellular energy, providing the metabolic power needed for the production of viral genomes and proteins during the viral replication cycle. Phosphatidylcholine is a primary building block of membrane-associated viral replicase complexes, used for robust replication and envelope formation in various RNA viruses [31]. Phosphatidylglycerol is a phospholipid component of pulmonary surfactant that can inhibit the binding of viruses to host cells, thus exerting antiviral effects [39]. Our study suggests that in PLWH who have co-infection with SARS-CoV-2, there may be alterations in the metabolism of TG, PC, and PG lipids, appearing as increased levels of TG and PC, and decreased levels of PG. This could be a result of the viral interactions or related to the abnormal activation of the immune system. The interrelated effects of lipids discovered through correlation analysis may be one of the mechanisms for disease progression on COVID-19 in HIV patients. Understanding the main pathways and lipid molecules involved in the reshaping of lipid metabolism induced by the virus may provide new insights into the pathogenesis and potential therapeutic targets for HIV patients who are co-infected with SARS-CoV-2. To sum up, our data indicate that the metabolism of TG, PC, and PG lipids plays a significant role in HIV co-infection with COVID-19 and is an important characteristic of disease occurrence. Changes in lipid content and chain length in plasma from the two groups may serve as biological markers for the development of disease and could potentially be used to distinguish between HIV patients co-infected with SARS-CoV-2 and those without SARS-CoV-2 infection.

This study has several limitations. Due to the limited sample size and the lack of longitudinal cohort analysis, there has not been a systematic investigation into the disease progression of HIV co-infection with COVID-19. This study did not include an HIV-negative patient group, making it impossible to accurately determine whether the significantly different metabolites are related to HIV infection. In addition, it is not known whether the changes in metabolic characteristics in HIV and SARS-CoV-2 co-infection are related to the intestinal microbial translocation caused by systemic inflammatory activation in HIV/AIDS patients. To elucidate these issues, future studies should incorporate healthy control groups. In this study, several potential biomarkers and changes in metabolic molecules were identified, but causality testing has not been conducted. Given the nature of this observational study, without further evidence, it is not possible to establish causal inferences. In light of the findings from this study, our subsequent research endeavors should aim to encompass larger cohorts of individuals with HIV who are also affected by COVID-19, and methodically gather longitudinal data at various stages of the disease trajectory to substantiate these outcomes. Furthermore, the omics methodologies utilized in this investigation could be broadened to encompass scenarios of HIV co-infection with other pathogens.

In summary, in this study, alterations were identified in the plasma metabolites associated with disease onset in individuals with HIV co-infected with COVID-19 through metabolomics, significant disruptions were observed in lipid metabolism, and various differential metabolites were identified to distinguish between HIV patients who are negative and positive for COVID-19. Among these, metabolites related to vitamin K1 are potential biomarkers, and changes in TG, PC, and PG lipids appear to be the main metabolic features of HIV co-infection with COVID-19. Our research demonstrates the added value of incorporating plasma metabolomics into a multi-omics analysis to assess the immunopathological biology and clinical processes of HIV co-infection with COVID-19.

## Figures and Tables

**Table 1:** Clinical characteristics of patients enrolled in this study.

Parameters	HIV/AIDS Patients Co-Infected with SARS-CoV-2 (n = 30)	HIV/AIDS Patients Without SARS-CoV-2 Infection (n = 30)	*p* Value
Male Gender (%)	93.33	86.67	0.389
Age (years)	51.37 ± 17.19	45.00 ± 12.42	0.106
HIV RNA (10^6^ copies/mL)	0.63 ± 1.38	0.58 ± 0.84	0.835
On ART (%)	56.67	36.67	0.121
CD4+ T (cells/μL)	81.27 ± 144.10	63.78 ± 104.50	0.621
CD8+ T (cells/μL)	493.20 ± 439.90	467.30 ± 287.90	0.921
CD4+ T/CD8+ T cell count	0.19 ± 0.29	0.18 ± 0.27	0.860
WBC (10^9^/L)	5.89 ± 2.73	6.42 ± 3.19	0.491
Hb (g/L)	114.40 ± 25.18	114.2 ± 24.88	0.980
N (10^9^/L)	4.37 ± 2.50	4.91 ± 3.07	0.459
L (10^9^/L)	0.88 ± 0.62	0.86 ± 0.58	0.877
M (10^9^/L)	0.49 ± 0.37	0.46 ± 0.28	0.649
SaO_2_ (%)	98.41 ± 2.46	97.30 ± 2.56	0.538

*p* value < 0.05 is considered statistically significant. Values are mean ± SD. WBC, white blood cell; Hb, hemoglobin; N, neutrophil; L, lymphocytes; M, monocyte; SaO_2_: oxygen saturation.

**Table 2:** Statistical analysis of differential metabolites between two groups of positive ion mode.

Metabolites	VIP Value	Fold Change	*p* Value
Fenpropidin	19.497	1.135	<0.001
L-carnitine	17.529	1.271	0.017
Pro-ile	11.623	2.443	0.036
2,4,6-tri-tert-butylaniline	8.859	1.144	<0.001
Picrotin	8.251	76.152	0.006
Phytosphingosine	5.745	1.262	<0.001
Lauryldimethylamine oxide	4.605	1.189	<0.001
Ng,ng-dimethyl-l-arginine	4.136	1.429	0.030
Daunorubicin	3.513	3.499	0.003
Myristamine oxide	3.329	1.248	<0.001
Heptadecasphinganine	3.115	0.329	0.001
Prolintane	3.096	1.115	<0.001
C17-sphinganine	3.026	0.333	0.002
Arachidoyl ethanolamide	2.964	1.098	<0.001
(r)-(+)-arachidonyl-1′-hydroxy-2′-propylamide	2.848	2.720	<0.001
Thioetheramide-PC	2.770	0.792	0.050
Oxaprozin	2.765	11.136	0.008
D-erythro-imidazolylglycerol phosphate	2.559	0.771	0.043
Myristoyl ethanolamide	2.283	1.123	<0.001
Phosphorylcholine	2.215	0.787	0.038
Hydroquinone	2.166	1.339	0.033
Palmitamide	1.976	1.155	<0.001
Triclabendazole	1.890	1.327	0.040
N,n-dimethyldodecylamine	1.857	1.367	0.004
1-stearoyl-2-arachidonyl-sn-glycero-3-phosphocholine	1.657	0.722	0.038
N-stearoylsphinganine	1.626	0.930	0.001
Ddao	1.608	1.204	<0.001
Gardenin b	1.604	1.307	0.007
Thr-Ala-Arg	1.591	1.334	0.014
N,n-dimethyltetradecylamine	1.531	1.251	0.004
Doxorubicin	1.519	4.255	0.003
Benzyl alcohol	1.437	0.757	0.044
Diethanolamine	1.425	1.586	0.006
(1r,5s)-9-methyl-9-azabicyclo[3.3.1]nonan-3-amine	1.346	2.131	0.029
Ethosuximide	1.253	1.289	0.027
Hydralazine	1.228	0.740	0.006
Sphingosine	1.190	1.099	0.001
N-octanoylsphingosine	1.165	2.461	0.001
N-myristoylsphinganine	1.164	1.172	<0.001
Isoeugenyl acetate	1.163	1.341	0.036
Pro-hyp	1.133	1.518	0.036
4′-hydroxychalcone	1.091	0.711	0.038
Ethylmorphine	1.038	3.202	0.001
6h-dibenzo[b,d]pyran,3-(1,1-dimethylbutyl)-6a,7,10,10a-tetrahydro-6,6,9-trimethyl-, (6ar,10ar)-	1.025	1.476	<0.001
N-methyldodecylamine	1.013	1.184	0.003
5-androstene-3.beta.,17.beta.-diol	1.012	1.497	<0.001
2-imidazolidinethione	1.012	1.221	0.035

*p* value < 0.05 is considered statistically significant. VIP, variable importance in the projection.

**Table 3:** Statistical analysis of differential metabolites between two groups of negative ion mode.

Metabolites	VIP Value	Fold Change	*p* Value
Gly-His-Lys	8.783	1.182	0.001
Hydroquinidine	8.553	1.159	0.003
Thymol-beta-d-glucoside	7.115	1.143	0.011
Dl-lactate	5.896	0.774	0.028
L-pyroglutamic acid	4.410	0.723	0.045
Ostruthin	4.106	1.174	0.001
Embelin	3.971	1.226	0.012
D-xylose	3.463	1.288	0.025
3,4-dihydroxymandelic acid	3.403	1.333	0.025
Kendomycin	3.316	3.534	<0.001
D-glucosamine 1-phosphate	3.093	3.964	0.002
Rauwolscine	2.731	1.200	0.038
Methylphosphonic acid	2.611	1.236	0.025
Amastatin	2.143	1.213	0.001
Sepiapterin	2.075	1.717	<0.001
L-cysteinesulfinic acid	2.024	3.714	0.006
5-heptenoic acid, 7-[(1r,2r,3s,5s)-2-[(1e,3s)-3-(2,3-dihydro-1h-inden-2-yl)-3-hydroxy-1-propen-1-yl]-3-fluoro-5-hydroxycyclopentyl]-, (5z)-	2.007	1.238	0.019
Aminocyclopyrachlor	1.846	0.611	0.015
D-gluconate	1.762	1.532	0.003
3-[(2,6-dihydroxy-3,4-dimethylphenyl)methyl]-2,4-dihydroxy-6-methylbenzaldehyde	1.677	0.625	<0.001
Pyruvaldehyde	1.495	1.530	<0.001
Dehydrotumulosic acid	1.486	0.723	0.019
17,20-dimethylprostaglandin f1.alpha.	1.449	0.689	0.003
1-naphthol	1.337	1.566	0.025
Vitamin k1	1.231	0.736	0.043
Lanosterol	1.207	0.696	0.010
2-arachidonoyl-1-stearoyl-sn-glycero-3-phosphoethanolamine	1.199	0.739	0.022
3-furancarboxylic acid, tetrahydro-4-methylene-2-octyl-5-oxo-, (2r,3s)-rel-	1.186	0.781	<0.001
Beta-elemonic acid	1.157	0.654	0.002
5′-o-acetyladenosine	1.029	4.974	0.013

*p* value < 0.05 is considered statistically significant. VIP, variable importance in the projection.

**Table 4:** Statistical analysis of differential lipids between two groups.

Lipid	Class	VIP Value	Fold Change	*p* Value
PC (23:0_11:2) +H	PC	6.967	1.178	0.007
PG (31:2e) +NH_4_	PG	6.323	0.813	0.001
PG (18:0_14:0) +H	PG	3.560	0.829	0.001
PC (16:0_16:1) +HCOO	PC	2.871	1.494	0.001
PS (18:0_20:4)-H	PS	2.644	0.688	0.044
TG (16:0_18:3_18:3) +H	TG	1.871	1.380	0.028
PC (14:0_18:2) +HCOO	PC	1.765	1.413	0.028
TG (6:0_10:3_10:4) +NH_4_	TG	1.493	0.853	0.001
TG (18:1_18:1_22:6) +NH_4_	TG	1.448	0.656	0.035
PG (16:0e) +NH_4_	PG	1.390	0.744	0.000
PE (14:0e_22:3) +Na	PE	1.356	0.290	0.005
TG (4:0_10:1_10:3) +Na	TG	1.274	0.860	0.001
PE (16:0_22:6)-H	PE	1.127	1.401	0.019
PC (17:1_20:4)-CH_3_	PC	1.112	1.212	0.033
Hex1Cer(t18:0_20:5) +HCOO	Hex1Cer	1.035	0.636	0.011
PG (18:0_19:0) +H	PG	1.018	0.534	0.004

*p* value < 0.05 is considered statistically significant. VIP, variable importance in the projection; PC, phosphatidylcholine; PG, phosphatidylglycerol; PS, phosphatidyl-serine; TG, triglycerides; PE: phosphatidyl ethanolamine.

## Data Availability

The original contributions presented in this study are included in the article. Further inquiries can be directed to the corresponding authors.
